# Increased oxytocin release precedes hyponatremia after pituitary surgery

**DOI:** 10.1007/s11102-020-01121-4

**Published:** 2021-01-28

**Authors:** Paul Eugène Constanthin, Nathalie Isidor, Sophie de Seigneux, Shahan Momjian

**Affiliations:** 1 Department of Neurosurgery, Hôpitaux Universitaires de Genève (HUG), Geneva, Switzerland; 2 Faculty of Medicine, Université de Genève (UNIGE), Geneva, Switzerland; 3 Clinical Investigation Unit, Clinical Research Center, University of Geneva, Hôpitaux Universitaires de Genève (HUG), Geneva, Switzerland; 4 Department of Nephrology, Hôpitaux Universitaires de Genève (HUG), Geneva, Switzerland

**Keywords:** Neurosurgery, Oxytocin, Hyponatremia, Pituitary gland

## Abstract

**Purpose:**

The syndrome of inappropriate secretion of antidiuretic hormone (SIADH) is a well-known complication of transsphenoidal pituitary surgery, related to inappropriate secretion of arginine vasopressin (AVP). Its diagnosis is based on hyponatremia, with a peak of occurrence around day 7 after surgery and, to date, no early marker has been reported. In particular, copeptin levels are not predictive of hyponatremia in this case. Oxytocin (OXT) is secreted into the peripheral blood by axon terminals adjacent to those of AVP neurons in the posterior pituitary. Besides its role in childbirth and lactation, recent evidences suggested a role for OXT in sodium balance. The contribution of this hormone in the dysnatremias observed after pituitary surgery has however never been investigated.

**Methods:**

We analyzed the urinary output of OXT in patients subjected to transsphenoidal pituitary surgery.

**Results:**

While OXT excretion remained stable in patients who presented a normonatremic postoperative course, patients who were later diagnosed with SIADH-related hyponatremia presented with a significantly increased urinary secretion of OXT 4 days after surgery.

**Conclusion:**

Taken together, these results show for the first time that urinary OXT output remains normally stable after transsphenoidal pituitary surgery. OXT excretion however becomes abnormally high on or around 4 days after surgery in patients later developing hyponatremia, suggesting that this abnormal dynamics of OXT secretion might serve as an early marker for transsphenoidal surgery-related hyponatremia attributed to SIADH.

**Supplementary Information:**

The online version contains supplementary material available at 10.1007/s11102-020-01121-4.

## Introduction

Transsphenoidal surgery of the pituitary gland is a well-established treatment for pituitary gland tumors. While the different technical aspects of this surgery are now well documented, it is not deprived of risks and complications such as hyponatremia, which might occur in up to more than a third of the patients as was reported in the literature [1–6]. One of the cause of hyponatremia is the syndrome of inappropriate secretion of antidiuretic hormone (SIADH), which is characterized by an inappropriately increased release of AVP, leading to an abnormal retention of water, finally resulting in potentially life-threatening hyponatremia and hypo-osmolality [7]. AVP is produced in the hypothalamus and secreted through nervous terminals running to the posterior part of the pituitary gland. Interestingly, oxytocin (OXT), another hormone, is secreted in the bloodstream through nervous terminals adjacent to the ones of AVP in the posterior hypophysis [8]. Unlike AVP, however, the secretion of OXT after transsphenoidal surgery has never been studied previously and nothing is known about the post-surgical dynamics of such secretion. This might be of relevance as several functions have been identified for OXT in the last decade. Indeed, recent studies have suggested a role for this hormone in social behaviors such as trust [9–13] or couple formation [14, 15] as well as in metabolism [16–19]. Another fact of particular importance is that OXT has been reported to play a role in sodium balance [20, 21]. This final role thus might be of importance in the case of transsphenoidal surgery-induced hyponatremia. Indeed, abnormal OXT secretion concomitant to SIADH might also contribute to sodium imbalance and therefore to hyponatremia.

In this study, we analyzed the urinary output of OXT in patients subjected to transsphenoidal pituitary surgery on the day of transsphenoidal pituitary surgery (D0) as well as on day 1 (D1), day 4 (D4) and day 7 (D7) after the surgery.

## Materials and methods

Patients that were elected for transsphenoidal surgery of the pituitary gland for pituitary micro- and macro-adenomas or Rathke’s cleft cysts were prospectively enrolled in the study at our center between November 2015 and February 2018. Patients’ levels of sodium and osmolality in both blood and urine as well as fluids intakes and urine volumes were followed daily during hospitalization and the presence of SIADH was diagnosed by the appearance of an increased urinary sodium concentration, hyponatremia (<136 mmol/l) and blood hypo-osmolality compared to urine. All patients without SIADH were discharged on D7 post-surgery while patients having been diagnosed with SIADH were treated accordingly with hydric restriction until correction of hyponatremia. If patients had a postoperative basal cortisol level on D4 below 500 nmol/l, they were maintained on 5 mg of prednisone per day to cover the time period until a follow-up cortisolemia evaluation in the following weeks.

### Biological samples

Biological samples were analyzed as previously described [22]. Urine samples were obtained after an overnight fasting period on the day of the surgery and on days 1, 4 and 7 after the surgery. Samples were collected in prechilled plastic ethylenediaminetetraacetic acid tubes containing a proteinase inhibitor (Trasylol; Bayer, Leverkusen, Germany) and then centrifuged at 1300 × *g* for 10 min at 4 °C. Samples were stored at −80 °C until analysis. Samples (0.5 ml) were kept at −20 °C until extraction using LiChroprep Si60 (Merck, Darmstadt, Germany) heat-activated at 700 °C for 3 h. Twenty milligrams of LiChroprep Si60 in 1 ml of distilled water was added to the sample, mixed for 30 min, washed twice with distilled water and 0.01 N HCl and eluded with 60% acetone. Extraction recovery was in the range 85–90%; data were not corrected for recovery. The lyophilised extract was then submitted to assay OXT using highly sensitive and specific radioimmunoassays (RIAgnosis, Munich, Germany). Although mainly used on plasma so far, the assay was strictly standardized and validated in many animal and human studies using a wide variety of stimuli (hypertonicity, parturition, lactation, stress, etc.) to reliably detect the bioavailable neuropeptides. Anti-OXT were raised in rabbits; as tracers, ^125^I-labelled neuropeptides (Perkin Elmer, Boston, MA, USA) were used. Assay sensitivities are in the 0.5-pg range, depending on the age of the tracers; cross-reactivities with related peptides, including AVP and desmopressin, are <0.7% and intra- and inter-assay variabilities are <10%. Using this assay, the basal urinary OXT levels (standardized to urinary creatinine) obtained in our patients are comparable to the basal values found in other studies using other assays [23, 24].

### Study design and ethical approval

Data were collected in a prospective way. An informed consent was obtained from all patients included in the study. The ethical approval regarding blood and urinary samples and their analyzes and patients’ data management was given by the local ethics committee (ethics committee number: 14-153).

### Role of the funding source

The funding source did not take part in any way during study design, in the analysis and interpretation of data, in the writing of the report or in the decision to submit the paper for publication. The corresponding author (SM) had full access to all the data in the study and had final responsibility for the decision to submit for publication.

### Patients and public involvement

Patients were not involved in any part of the research except for obtaining informed consent and biological samples.

### Statistical analyses

GraphPad Prism version 8 was used for statistical analysis. Descriptive statistics were expressed as median and quartiles (Q1; Q3) in the tables. Both groups (normonatremic and hyponatremic patients) were compared using unpaired *t*-test and Fisher’s exact test. For the analyses of urinary OXT, OXT values (standardized to creatinine excretion) on each day (day 0, day 1, day 4 and day 7) were compared between groups using an unpaired *t*-test. The evolution of OXT values was then compared for all patients and for each group (normonatremic and hyponatremic patients) by repeated ANOVA with a Bonferroni post-test. The ratios of OXT levels between successive days (D1/D0, D4/D1 and D7/D4) were finally compared in both normonatremic and hyponatremic patients using a two-way ANOVA with a Bonferroni post-test. Blood absolute values and urinary excretion (standardized to creatinine excretion) of sodium were compared between the two groups at post-operative days 1, 4 and 7 using a two-way ANOVA with a Bonferroni post-test. A *p* value lower than 0.05 was considered statistically significant.

## Results

### Groups

Twenty-one patients, 11 women (52.4%) and 10 men (47.6%) with a median age of 49 (41.75; 62) years old were prospectively recruited between November 2015 and February 2018 based on MRI imaging. Of those patients, seven were diagnosed with SIADH during hospitalization. They were treated in accordance with intra-hospital guidelines until normalization of sodium levels as well as urinary output and patients were discharged without further complication. Twenty patients presented with a pituitary adenoma (18 macros and 2 micros) and 1 patient presented with a Rathke’s cleft cyst.

Preoperatively, both groups (hyponatremic and normonatremic) were significantly different for lesion volume and Knosp grade (with patients later developing hyponatremia presenting with larger tumors resulting in a higher proportion of Knosp grades 3 and 4 in this group). They were however not significantly different for age, gender, ethnicity, baseline weight, baseline arterial blood pressure (BP), comorbidities, pathology (proportion of micro- or macro-adenomas and cysts, proportion of secreting and non-secreting tumors) or treatments. Postoperatively, both groups showed no statistical difference in the occurrence of transient diabetes insipidus (DI), basal cortisol values, hypocortisolemia necessitating maintained corticoid replacement therapy or average daily urinary output volume from D0 until D7 (Table 1). The patients who presented with transient DI were all treated with one dose of desmopressin on D1. Two of these patients (one in the normonatremic group and one in the hyponatremic group) received an additional dose of desmopressin on D4. Of note, all these doses of desmopressin happen to have been given in the evening or night of D1 and D4 while urinary sampling and hence measurement of urinary OXT was done in the morning of D1 and D4, i.e. always before desmopressin administration.

**Table 1:** Comparison of hyponatremic and normonatremic patients

	Normonatremic (*n* = 14)	Hyponatremic (*n* = 7)	*p* Value	Statistical test
Age (y.o.)	51 (45; 60)	40 (38; 66.5)	0.7325	Unpaired *t*-test
Gender (female)	57.1%	42.9%	0.6594	Fischer’s exact test
Weight (kg)	81.8 (69; 89)	73.8 (70.8; 81.1)	0.7317	Unpaired *t*-test
Systolic BP (mmHg)	133 (121; 138)	123 (118; 135)	0.5875	Unpaired *t*-test
Diastolic BP (mmHg)	84 (75; 93)	74 (68; 77)	0.1	Unpaired *t*-test
High BP	2	1	>0.999	Fischer’s exact test
Hypercholesterolemia	3	1	>0.999	Fischer’s exact test
Diabetes	1	1	>0.999	Fischer’s exact test
Preoperatvie hypothyroidism	1	1	>0.999	Fischer’s exact test
Preoperative corticoid replacement therapy	4	2	>0.999	Fischer’s exact test
Psychoactive medication	4	1	0.6244	Fischer’s exact test
Ethnicity (caucasian; other)	12; 2	6; 1	>0.999	Fischer’s exact test
Pathology (adenoma; cyst)	13; 1	7; 0	>0.999	Fischer’s exact test
Hormonal secretion from adenoma (yes; no)	6; 7	0; 7	0.0515	Fischer’s exact test
Lesion volume (mm^3^)	3332 (864; 4080)	9660 (4132; 21,994)	0.0291*	Unpaired *t*-test
Knosp grade (1–2; 3–4)	11; 2	2; 5	0.0223*	Fischer’s exact test
Postoperative basal cortisol (nmol/l)	338 (235; 400)	327 (267; 367)	0.8493	Unpaired *t*-test
Hypocortisolemia necessitating maintained corticoid replacement therapy (yes; no)	14; 0	7; 0	0.6126	Fischer’s exact test
Transient DI (yes; no)	2; 12	4; 3	0.1196	Fischer’s exact test
Average daily urinary output volume during hospitalization (ml)	917 (756; 1457)	1279 (862; 1344)	0.4644	Unpaired *t*-test

Significant *p* values are shown in bold

Continuous values are expressed as median and quartiles (Q1; Q3)

*p < 0.05

### Urinary oxytocin levels remain stable after pituitary transsphenoidal surgery in normonatremic patients

**Fig. 1:**
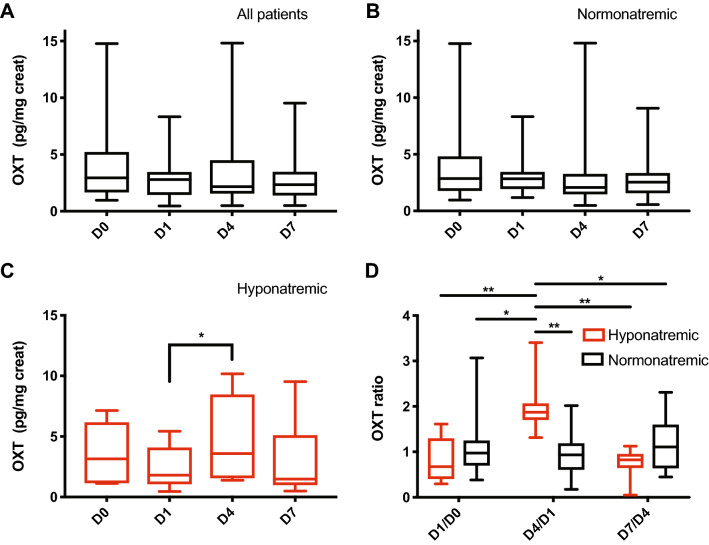
Urinary OXT levels show a perturbed pattern after pituitary surgery in hyponatremic patients. (**a**) Evolution of OXT urinary secretion in all patients between D0, D1, D4 and D7 (*n* = 20 patients). (**b**) Evolution of OXT urinary secretion between D0, D1, D4 and D7 in normonatremic patients (*n* = 13 patients). (**c**) Evolution of OXT urinary secretion between D0, D1, D4 and D7 in hyponatremic patients (*n* = 7 patients; D4 vs D1: 95% CI −4.413 to −0.08275, *p* = 0.0428). (**d**) Comparison of the ratios of urinary secretion of OXT (value on D1 relative to the value on D0, value on D4 relative to the value on D1 and value on D7 relative to the value at D4) between both groups (normonatremic (n): *n* = 13 patients, hyponatremic (h): *n* = 7 patients; (D4/D1)h vs (D4/D1)n: 95% CI 0.2462–1.892, *p* = 0.003). Repeated measures one-way ANOVA with Bonferroni post-test (**a**–**c**) and two-way ANOVA with Bonferroni post-test (**d**); median and quartiles; *p < 0.05, **p < 0.01

We compared the urinary levels of OXT (standardized to urinary creatinine) in the patients on D0, D1, D4 and D7 and found no statistical difference between the normonatremic and hyponatremic groups (due to a missing urinary creatinine value, one patient of the normonatremic group was excluded from the analyzes) (Table 2). We then analyzed the evolution of OXT secretion after surgery by comparing the OXT values across the different successive days. When the results of all patients were analyzed together, we did not observe any significant change of OXT secretion on D0, D1, D4 and D7 (Fig. 1a). Such a stability of OXT urinary excretion was also observed in patients remaining normonatremic later on (Fig. 1b). Taken together, these results suggest that OXT secretion remains stable after transsphenoidal pituitary surgery in patients having a normal postoperative natremic course.

**Table 2:** Summary and comparison of urinary OXT values (median and quartiles (Q1; Q3))

	All patients together (*n* = 21)	Normonatremic patients (*n* = 14)	Hyponatremic patients (*n* = 7)	*p* Value (between normonatremic and hyponatremic)
D0 (pg/mg creat)	2.93 (1.71; 4.4)	2.86 (1.81; 3.99)	3.16 (1.39; 4.89)	0.781
D1 (pg/mg creat)	2.8 (1.61; 3.44)	2.84 (2.16; 3.43)	1.8 (1.08; 3.61)	0.2174
D4 (pg/mg creat)	2.16 (1.56; 3.9)	2.07 (1.56; 3.2)	3.6 (1.72; 7.11)	0.1569
D7 (pg/mg creat)	2.34 (1.45; 3.24)	2.55 (1.65; 3.12)	1.49 (1.1; 4.03)	0.4045

Unpaired *t*-test

### The pattern of oxytocin secretion is perturbed in patients with SIADH

We were then interested to know whether the dynamics of OXT secretion was altered in patients later presenting with hyponatremia. Interestingly, while OXT excretion remained stable in normonatremic patients, patients later diagnosed with SIADH presented a significant increase of urinary excretion of OXT on D4 compared to D1, suggesting the concomitant existence of a syndrome of inappropriate secretion of OXT (SIOXT) in these patients (D4 vs D1: 95% CI −4.413 to −0.08275, *p* = 0.0428) (Fig. 1c). This was strengthened when calculating the ratios of OXT secretion between the successive days (D1/D0, D4/D1 and D7/D4), where a significant difference appeared between the normonatremic (n) and hyponatremic (h) patients, with a specific increase of the OXT secretion ratio on D4 in the latter group only [(D4/D1)h vs (D1/D0)h: 95% CI −2.173 to −0.2972, *p* = 0.0025; (D4/D1)h vs (D1/D0)n: 95% CI −1.757 to −0.1114, *p* = 0.0147; (D4/D1)h vs (D4/D1)n: 95% CI 0.2462–1.892, *p* = 0.003; (D4/D1)h vs (D7/D4)h: 95% CI 0.3479–2.224, *p* = 0.0015; (D4/D1)h vs (D7/D4)n: 95% CI 0.03762–1.683, *p* = 0.0334] (Fig. 1d and Table 3). Of note, a decrease of OXT secretion on D1 compared to D0, leading to a median D1/D0 OXT ratio of 0.67, can still be discerned in the hyponatremic patients (see Fig. 1c) although this change did not reach statistical significance.

**Table 3:** Comparison of OXT dynamics between hyponatremic and normonatremic patients (median and quartiles (Q1; Q3))

	OXT in normonatremic patients (*n* = 13)	OXT in hyponatremic patients (*n* = 7)	*p* Value
OXT ratio D1/D0	0.98 (0.71; 1.21)	0.67 (0.45; 1.03)	>0.999
OXT ratio D4/D0	0.8 (0.37; 1.11)	1.36 (1.04; 2.05)	0.3046
OXT ratio D7/D0	0.72 (0.42; 1.6)	0.82 (0.51; 1.98)	0.8142
OXT ratio D4/D1	0.94 (0.64; 1.17)	1.87 (1.77; 3.03)	0.0025**
OXT ratio D7/D1	0.76 (0.44; 1.24)	1.64 (1.01; 1.98)	0.409
OXT ratio D7/D4	1.11 (0.66; 1.36)	0.82 (0.68; 0.92)	>0.999

Significant *p* values are shown in bold

Two-way ANOVA with Bonferroni post-test

**p < 0.01

To summarize, these results show for the first time that OXT secretion after pituitary transsphenoidal surgery is altered in patients presenting with SIADH, with a dynamic release of OXT on D4. This suggests that pituitary transsphenoidal surgery might also result in a SIOXT.

### SIOXT preceded hyponatremia in SIADH patients

**Fig. 2:**
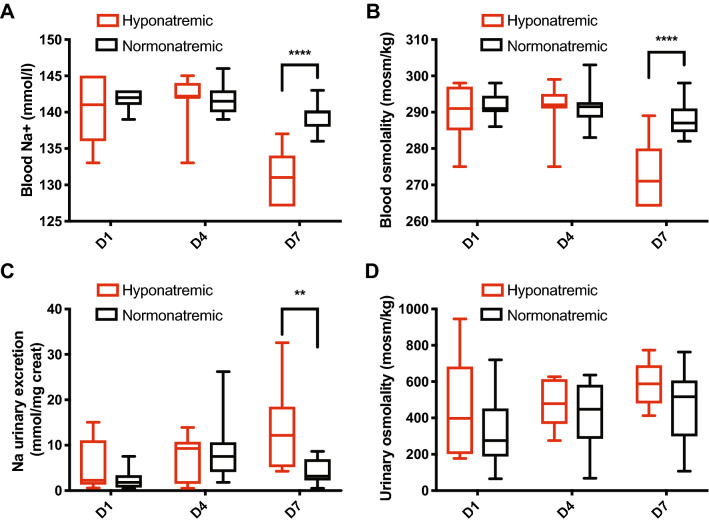
Blood sodium and osmolality and as well as urinary sodium excretion showed abnormal values only on D7 after surgery. (**a**) Comparison of the blood concentration of sodium at D1, D4 and D7 between normonatremic and hyponatremic patients (normonatremic: *n* = 13 patients, hyponatremic: *n* = 7 patients; 95% CI for the significant value −13.18 to −3.982, *p* < 0.0001). (**b**) Comparison of the blood osmolality at D1, D4 and D7 between normonatremic and hyponatremic patients (normonatremic: *n* = 13 patients, hyponatremic: *n* = 7 patients; 95% CI for the significant value −21.68 to −8.008, *p* < 0.0001). (**c**) Comparison of the urinary excretion of sodium at D1, D4 and D7 between normonatremic and hyponatremic patients (normonatremic: *n* = 13 patients, hyponatremic: *n* = 7 patients; 95% CI for the significant value −11.53 to −5.182, *p* = 0.002). (**d**) Comparison of the urinary osmolality at D1, D4 and D7 between normonatremic and hyponatremic patients (normonatremic: *n* = 13 patients, hyponatremic: *n* = 7 patients). Two-way ANOVA (**a**–**d**) with Bonferroni post-test; median and quartiles; **p < 0.01, ****p < 0.0001

SIADH is characterized by a pathological retention of free water, leading to hypo-osmolality and hyponatremia. SIADH-induced hyponatremia mostly occurs only on D7 post-surgery and patients often remain in the hospital until that time. We therefore measured urinary sodium excretion and blood natremia on D1, D4 and D7 to evaluate whether SIOXT might precede SIADH-induced hyponatremia. Hyponatremic patients showed pathological sodium and osmolality values in the blood on D7 compared to normonatremic patients but no significant difference were observed on D1 and D4 (Fig. 2a, b). Interestingly, sodium excretion was significantly higher on D7 in hyponatremic patients although urine osmolality did not differ between groups (Fig. 2c, d). There was no significant difference between the two groups regarding BP or patient’s weight during inpatient follow-up (Supplementary Fig. 1a–c).

These results show for the first time that a pathologically increased OXT secretion precedes SIADH-induced hyponatremia after pituitary transsphenoidal surgery. This suggests a potential role for OXT as an early marker of this syndrome. Moreover, we show that urinary sodium excretion is increased on D7, suggesting a pathological role for SIOXT in postoperative hyponatremia.

## Discussion

In this study, we measured the evolution of OXT urinary excretion after pituitary transsphenoidal surgery, hypothesizing that OXT release would be influenced by the surgical procedure. Interestingly, while no specific dynamics of OXT excretion were noted when patients remained normonatremic, we observed that urinary output of OXT significantly increased 4 days after surgery in patients later diagnosed with SIADH-related hyponatremia. Furthermore, this abnormal OXT excretion preceded hyponatremia, suggesting that the abnormal dynamics of OXT secretion might serve as an early marker for the diagnosis of transsphenoidal surgery-related hyponatremia.

Besides anterior pituitary insufficiency, abnormal sodium balance due to posterior pituitary dysfunction are known to occur after transsphenoidal pituitary surgery. The role of AVP in such imbalances (being DI or SIADH) has been recognized. Our results suggest that patients with larger lesions might be at higher risk to develop SIADH. This seems to be in accordance with a previous observation made by Taylor and colleagues who reported a tendency, albeit not significant, for patients with macro-adenomas to develop hyponatremia more frequently than patients with micro-adenomas [5]. This may be explained by a more compressed and hence fragile pituitary gland that will be more prone to passively release AVP after surgical manipulation. Due to the impossibility to precisely measure AVP levels in the blood in the routine, diagnosis of AVP-related conditions remains based on the follow-up of water and sodium balance. SIADH-induced hyponatremia mostly occurs only on D7 post-surgery and patients often preferably remain in the hospital until that time for efficiency reasons in the insurance of adequate sodium monitoring and eventual hyponatremia management [3, 5, 7]. Therefore, early biomarkers are of particular interest to clinical practice and copeptin was considered as it appears to be a more stable and easier to measure surrogate marker of AVP that mirrors its secretion. Copeptin was recently reported as a promising marker of DI [25–27] but the same marker proved unreliable in the case of SIADH and there is, to date, no reliable early marker for this pathology despite active research in the field [28]. Of note, copeptin measured and analyzed serially as we did could have given similar findings and further studies should aim at comparing the predictive values of OXT and copeptin. Our study was designed to focus on the course of OXT for which there is overall much more experience with its simple assay in the urine than with urinary copeptin.

OXT is a neuropeptide hormone produced in the hypothalamus and released through the posterior part of the hypophysis in the bloodstream. OXT has been known to be implicated in lactation for a long time [17]. Interestingly, it has also been recently reported to be implicated in several other mechanisms, one of them being sodium regulation [20, 21]. Albeit abnormal secretion of AVP is accepted to be the main cause of water-sodium imbalances seen after pituitary surgery, there is no study to date that attempted to measure OXT levels before and in the days after transsphenoidal surgery.

In our study, we observed that patients that presented with normal natremia up to 1 week after surgery exhibited a stable release of OXT during the days after the surgery. This suggests that OXT secretion remains normally unaffected by transsphenoidal surgery. Of note, patients later diagnosed with SIADH-related hyponatremia, on the contrary, exhibited a significant increase of OXT secretion at 4 days after surgery. This is of particular importance as it suggests for the first time the existence of a SIOXT. Moreover, these patients showed decreased levels of OXT secretion on D1 compared to D0 and an increased proportion of transient DI on D1, albeit these differences remained statistically not significant in our group of patients. This association might suggest that a short period of pituitary “stunning” happens after pituitary surgery in the future hyponatremic patients, with transient DI in some of them. Indeed, as recently reviewed by de Vries et al. [29], SIADH can present as part of a biphasic pattern, i.e. following a transient bout of postoperative DI that typically occurs within 24–48 h after surgery and resolves in the next days. This transient DI episode is probably caused by pituitary “stunning” with a lack of AVP secretion. After this DI period, the reversibly injured pituitary gland starts to passively release stored AVP in the bloodstream, causing the SIADH phase. Thus, in our group of patients we explain the detection of a difference between D4 and D1, and not between D4 and D0 (the true baseline), by the apparent D1 OXT dip relative to D0. In a larger group of patients a difference between D4 and D0 might become detectable.

Furthermore, the existence of such a SIOXT can give rise to the hypothesis of a contributing role of OXT in the postoperative hyponatremia. In fact, OXT was reported to induce natriuresis in rats, partially through an increased release of atrial natriuretic peptide (ANP) [30–32]. Our findings of a higher natriuresis in hyponatremic patients on D7 could be the consequence of this latter effect and are potentially relevant clinically as, along with the experimental evidence, they may suggest a contributing role of SIOXT in the SIADH-associated hyponatremia, with an increased renal excretion of sodium related to an increased OXT secretion, thus aggravating the phenomenon by counteracting the previously reported antinatriuretic effects of AVP on the kidney [32, 33]. The likelihood of a hypothetical secondary causal contribution of OXT in the occurrence of hyponatremia is however reduced by the absence of any observed relative increase of urinary OXT on D7 in the hyponatremic patients. Nevertheless, the 3-day lag time between the elevation of OXT on D4 and the hyponatremia on D7 could be explained by the respective mechanisms of action of AVP and OXT leading to the excretion of water and sodium. If OXT is only a surrogate marker of SIADH, one can posit that the OXT axonal terminals passively release their neuropeptide at the same time than the AVP axonal terminals, starting early in the days after surgery, but, taking into account the mechanisms of action of AVP (namely an increased insertion but also transcription of water channels into the kidney tubes), the occurrence and nadir of hyponatremia could be delayed 3 days later. If OXT also contributes to hyponatremia, one can posit a chain of events passing through an induced secondary release of ANP [30, 34] that would also take some time to accentuate the delayed hyponatremia. This emphasizes the potential importance of the existence of SIOXT as it sheds a new light on the causes of the hyponatremia induced by transsphenoidal pituitary surgery, which warrants further research.

Another important finding of our study is the fact that abnormal OXT secretion preceded the appearance of sodium imbalance in hyponatremic patients. This suggests that SIOXT might accompany or precede SIADH, and hence mark or predict it. These results show for the first time a potential for OXT as a predictive biomarker for hyponatremia as abnormal OXT secretion was observed prior to any abnormality in sodium balance. Such a biomarker, if confirmed, would be of utmost interest for clinical practice as it could dramatically reduce the duration of the hospital stay or close outpatient follow-up by identifying patients at risk of sodium imbalance as early as 4 days after surgery, and further research is warranted in this direction. In particular, the predictive value of the D4/D1 OXT ratio should be further evaluated in larger cohorts. Finally, further research should also investigate on a larger scale the trend of a decrease of OXT secretion on D1 compared to D0, as the D1/D0 OXT ratio could appear as a biomarker of postoperative pituitary “stunning” and hence of risk of transient DI as well as of SIADH later on.

Of course, our study presents with limitations, the main one being the limited size of the studied population (21 patients in total). Further studies with larger patients’ cohorts are therefore required. The clinical applicability of our results is also limited by the lack of a medical grade method for determining OXT levels routinely with clinical accuracy and further development on OXT measurements is warranted to bring a practical and reliable method to the clinics. Moreover, we did not measure OXT secretion later than 7 days post-surgery and, therefore, we do not possess any information on OXT secretion later on. Although urinary OXT appears to correlate with plasma OXT (see Supplementary materials of 22), OXT secretion was only measured in the urine and the evolution of OXT levels in the plasma might have been informative too. Finally, we did not test the hypothesis whether the observed SIOXT might also play a direct role in natriuresis and hyponatremia, as this objective was beyond the scope of our study.

## Conclusion

In this study, we show for the first time that urinary OXT secretion remains normally stable after transsphenoidal pituitary surgery. However, we observed that OXT secretion becomes abnormally high in patients later diagnosed with SIADH-related hyponatremia, suggesting the existence of a SIOXT and a potential contributing role for this hormone in SIADH-associated hyponatremia. Finally, we show that SIOXT precedes SIADH-related pathological changes in sodium balance suggesting that OXT possesses the potential to act as an early biomarker of SIADH.

## Supplementary information

Below is the link to the electronic supplementary material.

Supplementary material 1 (PDF 391 kb)
Patients’ weight and arterial blood pressure (BP) did not show any statistically significant postoperative difference. (**a**) Comparison of the patients’ weight at D1, D4 and D7 between normonatremic and hyponatremic patients (normonatremic: *n* = 13 patients, hyponatremic: *n* = 7 patients). (**b**) Comparison of the patients’ diastolic BP at D1, D4 and D7 between normonatremic and hyponatremic patients (normonatremic: *n* = 13 patients, hyponatremic: *n* = 7 patients). (**c**) Comparison of the patients’ systolic BP at D1, D4 and D7 between normonatremic and hyponatremic patients (normonatremic: *n* = 13 patients, hyponatremic: *n* = 7 patients). Two-way ANOVA with Bonferroni post-test (**a**–**c**); median and quartiles (PDF 390 kb)

## Data Availability

Data are available from the corresponding author upon reasonable request.
