# Supplementary figures and images for: Increased oxytocin release precedes hyponatremia after pituitary surgery
Source: Pituitary. 2021 Jan 28;24(3):420–8. doi: 10.1007/s11102-020-01121-4 (PMC8119398; doi:10.1007/s11102-020-01121-4)

A

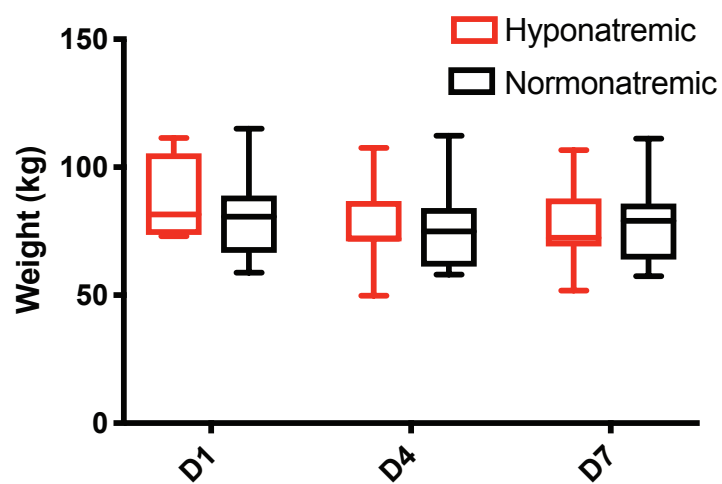

B

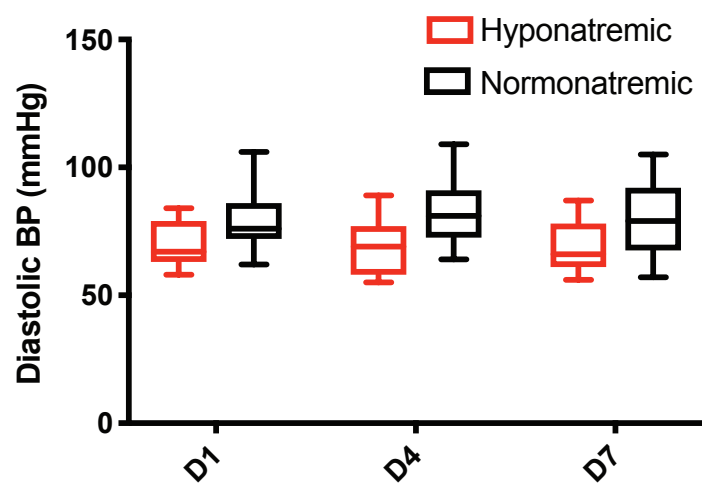

C

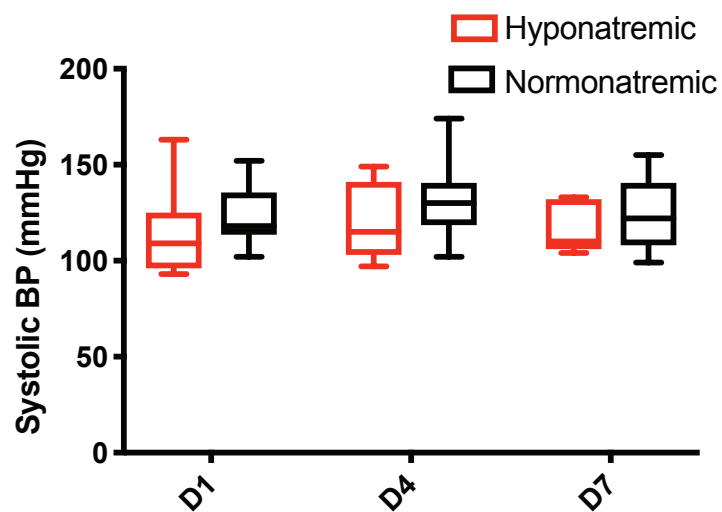

Supplement: Supplementary file 1 — Supplementary material 1 (PDF 391 kb) Patients’ weight and arterial blood pressure (BP) did not show any statistically significant postoperative difference. (a) Comparison of the patients’ weight at D1, D4 and D7 between normonatremic and hyponatremic patients (normonatremic: n = 13 patients, hyponatremic: n = 7 patients). (b) Comparison of the patients’ diastolic BP at D1, D4 and D7 between normonatremic and hyponatremic patients (normonatremic: n = 13 patients, hyponatremic: n = 7 patients). (c) Comparison of the patients’ systolic BP at D1, D4 and D7 between normonatremic and hyponatremic patients (normonatremic: n = 13 patients, hyponatremic: n = 7 patients). Two-way ANOVA with Bonferroni post-test (a–c); median and quartiles (PDF 390 kb) [file 11102_2020_1121_MOESM1_ESM.pdf]
